# A Phi29-based unbiased exponential amplification and genotyping approach improves pathogen detection in tick samples

**DOI:** 10.3389/fvets.2022.1025911

**Published:** 2022-11-07

**Authors:** Xi Zhang, Jingjing Chen, Pengfei Jiang, Heling Xu, Qi Zhang, Huan Zhang, Xiaohu Han, Zeliang Chen

**Affiliations:** ^1^Key Laboratory of Livestock Infectious Diseases, Ministry of Education, Shenyang Agricultural University, Shenyang, China; ^2^Department of Epidemiology, School of Public Health, Sun Yat-sen University, Guangzhou, China

**Keywords:** ticks, Phi29, *Rickettsia*, genotyping, pathogen detection and genotyping

## Abstract

Ticks are vectors for many infectious diseases, such as spotted fever group (SFG) rickettsioses and borreliosis, and are valuable in the study of pathogen ecology. Ticks have several growth stages that vary considerably in size; therefore, in most cases, DNA extracted from ticks is insufficient for subsequent studies, particularly for multiple pathogen screening and genotyping. Unbiased amplification of DNA from tick samples before analysis is a major requirement for subsequent ecological surveys and other studies. Phi29 DNA polymerase, an enzyme that exhibits strand displacement activity, can exponentially amplify DNA randomly, generating large quantities of DNA. In the present study, we developed a Phi29-based unbiased exponential amplification (PEA) assay to obtain sufficient tick DNA for genetic analysis. By using tick-borne pathogen detection and genotyping as a model, we tested and evaluated the feasibility of the assay. DNA was extracted from single ticks and subjected to PEA. The results showed that tick DNA could be amplified up to 10^5^ fold. The amplified products were successfully used for pathogen screening and genotyping. *Rickettsia* was successfully detected and genotyped in samples with amplified DNA from single ticks. Furthermore, we identified a new genotype of *Rickettsia* from ticks collected from Dandong city, Liaoning province, Northeast China. This PEA assay is universal and can be extended to other applications where the quantity of DNA is greatly limited.

## Introduction

The recent worldwide outbreaks of Q fever, Lyme disease, and Tick-borne spotted fever serve as a reminder of the global challenge presented by infectious diseases. Vector-borne diseases represent a significant proportion of emerging infectious diseases. Ticks, one of the most important vectors, are hosts for numerous pathogens causing zoonotic diseases (1–3). After mosquitoes, ticks are the most important vectors of human infectious diseases worldwide; compared with insects, ticks are more efficient in infecting, transmitting, and storing pathogens. Consequently, they pose a major threat to the health of humans, livestock, and poultry, as well as to property (4). Therefore, many investigations on pathogen ecology focus on tick surveillance to evaluate the transmission risk of infectious diseases and emerging infectious diseases in particular.

Ticks belong to the superfamily Ixodoidea within the phylum Arthropoda. The superfamily of ticks is further divided into the families Ixodidae, Argasidae, and Nuttalliellidae. Ticks are widespread, and mostly found in forests, grasslands, and bushes. The most medically relevant ticks belong to the Ixodidae family (5). The Asian longhorned tick (Haemaphysalis longicornis) is an ectoparasite that specializes in parasitizing and feeding on animal blood. These ticks have a high fecundity and can rapidly adapt to the environment. Their bites can damage an animal's epidermis, cause infection and death, and reduce the economics of livestock husbandry (6). Because ticks have a high frequency of host replacement and spawning, cross infections, which also affect humans, can occur infection of several zoonoses (7). Ticks have four life stages, namely eggs, larvae, nymphs, and adults (8). Free, unfed ticks are usually rather small however, the weight of adult female ticks can increase up to 300 times after feeding. Owing to their small size (9), only limited amounts of DNA can be extracted from unfed ticks, which makes it difficult to use them for subsequent analysis. Large amounts of DNA are frequently required for multiple pathogen screening and genotyping (10). The limited amount of DNA extracted from a single tick is always a critical obstacle to meeting the requirements of subsequent applications. To obtain sufficient DNA for subsequent use is essential for tick-borne disease surveillance.

Nucleic acids can be amplified with many polymerases; however, most of these show biased amplification, and result in short sequences. The Phi29 DNA polymerase, an enzyme that shows strand displacement activity, can exponentially amplify DNA randomly and unbiasedly, thereby generating large quantities of DNA (11). In particular, long DNA fragments and high DNA yields can be generated from very low amounts of starting material (12, 13). This makes the Phi29 polymerase an ideal tool to amplify DNA that is only available in trace amounts.

In the present study, we first tested the amplification efficacy of Phi29 on tick DNA. We then developed a new Phi29-based unbiased exponential amplification (PEA) assay and evaluated its application in pathogen detection and genotyping. PEA amplification reactions can be performed at a constant temperature of 30°C, providing more practical conditions for DNA amplification compared to traditional methods (14). DNA amplification can be performed on site to meet the needs of subsequent pathogen detection tests for veterinary clinical diagnosis. The Phi29 DNA polymerase is a mesophilic DNA polymerase cloned from the Bacillus subtilis phage phi29 (10). One characteristic of this enzyme is that the newly generated forward strand debranches from the original template. Owing to this feature, multiple copies can be generated from each location on the template in the first round of the reaction (15). This enables 1,000-fold amplification of trace amounts of DNA, which increases the detection rate without influencing the genotyping.

## Materials and methods

### Tick collection and morphological identification

Ticks were collected from goats and cattle raised in the village of Dandong city, Liaoning province, northeast China. They were manually removed from animal skin without damaging the ticks and then carefully placed into 75% ethanol (16). Tick samples are named after locations and serial number. All samples were sent to the laboratory of Shenyang Agricultural University and stored for further examination. All of these ticks were morphologically identified as *Haemaphysalis longicorni*s, the representative, marked features of which were as follows. The body was broadly oval or subcircular and yellowish-brown color, no eyes. The capitulum was short, blunt wedge-shaped. The basis capituli was rectangular with a distinct triangular strong posterior. Outer margin of whisker limb moderately convex, with a coniform present. The dentition of hypostome was 5/5. The female scutum was subcircular, the middle of scutum is broad, the male cornua was strong, the end is pointed. Punctuations on scutum were medium large, uniform distribution and dense. The cervical sulcus was short and curved, but the lateral sulcus was narrow and distinct. The spiracular plate was broadly oval. Feet of medium thickness with numerous setae (17). Ticks' growth cycle was divided into four stages, eggs, larva, nymph, and adults, larva with six legs, and nymph have no gonopore.

### Single tick DNA extraction and molecular identification

**Figure 1:**
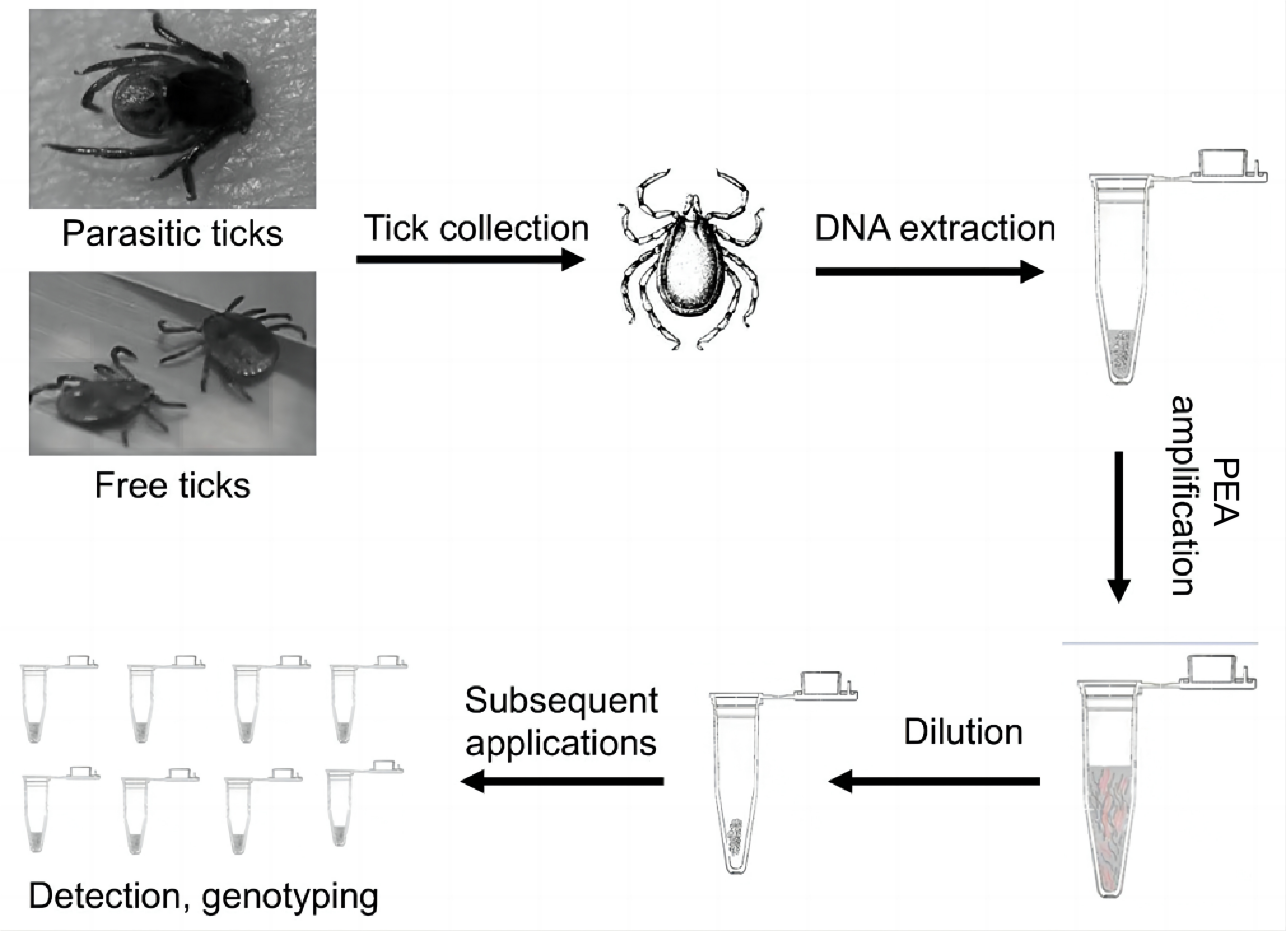
Schematic representation of PEA. Free or parasitic ticks were collected from natural environments or their hosts. After washing with 70% alcohol, the ticks as subjected to DNA extraction with genomic kits and amplified with PEA. The amplified DNA could be used for PCR detection, genotyping, or genomic analysis.

DNA was extracted using a DNA extraction kit (TransGen Biotech, ER201-01, Beijing, China) according to the manufacturer's protocol and stored at −20°C. The DNAsamples were named as Table 1. The tick DNA was subjected to PCR amplification. Twenty of the 186 positive samples (10.8%, selected according to the gender, host, and stage of the tick life cycle) were sent for sequencing. We designed a Phi29-based strategy to detect and genotype pathogens in individual ticks (Figure 1).

**Table 1:** Concentrations of PEA amplification of tick DNA and *Rickettsia* detecting proportion.

**Sample ID**	**DNA concentration (ng/ul)**		**Amplification index**		**Rickettsia detection**	
	**Tick DNA**	**PEA products**	**Tick DNA**	**Starting DNA**	**Tick DNA**	**PEA products**
EDG011	54.6	727.5	13.3	72.8	+	+
EDG015	43.7	1,006.9	23.0	100.7	+	+
EDG039	27.9	962.8	34.5	96.3	+	+
EDG044	31.2	890.2	28.5	89.0	+	+
EDG047	46.7	885.4	19.0	88.5	+	+
EDG149	19.9	785	39.4	78.5	+	+
EDG124	461.5	887.5	1.9	88.8	+	+
EDG135	382.8	825.6	2.2	82.6	+	+
SD002	31.3	692.3	22.1	69.2	+	+
PH008	438.7	875.5	2.0	87.6	+	+
LZG109	87.7	885.5	10.1	88.6	+	+
PH014	278.2	994.8	3.6	99.5	-	+
PH172	552.2	1,079.4	2.0	107.9	-	+
LZG102	148.3	941.2	6.3	94.1	-	+
EDG071	6.2	945.9	152.6	94.6	-	+
EDG103	6	889.8	148.3	89.0	-	+
SD010	50.3	1,021.9	20.3	102.2	-	-
KD219	121.7	410	3.4	41.0	-	-
KD213	23.3	1,351.5	58.0	135.2	-	-
EDG241	272.5	619	2.3	61.9	+	+

### Amplification of single tick DNA

To determine the optimal amplification of single tick DNA, we compared and optimized the amplification. The reaction time was set for 8 h, 16 h, and 24 h. The amplification was performed using the phi29 DNA Polymerase (#M0269L, NEB, USA)according to the manufacturer's instructions. A master mix was prepared by adding 2.5 μM random primer (NNNNNN), 1 μl DNA sample and 1 μl 10 × Phi29 DNA into a microcentrifuge tube, which was then vortexed and briefly centrifuged. The sample was placed in a metal bath at 95 °C for 3 min, and put on ice for 15 min. The final reaction mixture was set up by adding 0.5 μM Deoxynucleotide (dNTP) Solution Mix, 0.4 μg/μl BSA and 0.05 μg/μl phi29 DNA Polymerase to 10 μl. The reaction was performed at 30 °C for 16 h, followed by 10 min at 65 °C to stop the reaction. The concentration of the amplified DNA was measured, (By Thermo, Nanodrop 2000, USA) and the sample was diluted to the required final concentration to be used for subsequent analysis.

### *Rickettsia* detection and genotyping from single ticks

The *gltA, ompA and ompB* genes were PCR amplified from the diluted amplification product for *Rickettsia* detection using 2 × TaqMaster Mix polymerase (P112-01, Vazyme Biotech Co., Ltd, China) (18). The primers shown in Table 2. The PCR reactions were done as follows: 94 °C for 5 min denaturxation, followed by 37 cycles of 94°C for 30 s, 58°C for 40 s, and 72°C for 40 s (19). PCR products were sequenced by Sanger sequencing (Sangon Biotech Co., Ltd, Shanghai, China).and the generated sequences were compared with those from the database for phylogenetic analysis in GenBank. The phylogenetic tree was constructed based on the sequence distance method using the neighbor-joining (NJ) algorithms implemented in the Molecular Evolutionary Genetics Analysis (MEGA) 7 software.

**Table 2:** Primers used in the study.

**Gene**	**Primer/probe**	**Sequences (5^′^-3^′^)**
gltA	Cs-239	GCTCTTCTCATCCTATGGCTATTAT
	Cs-1069	CAGGGTCTTCGTGCATTTCTT
ompA	RcromA190-70	ATGGCGAATATTTCTCCAAAA
	RcromA190-71	GTTCCGTTAATGGCAGCATCT
ompB	ompB-1	TACTTCCGGTTACAGCAAAGT
	ompB-2	AAACAATAATCAAGGTACTGT

## Results

### Morphological characteristics and molecular identification of parasitic ticks

All ticks were morphologically and molecularly identified as H. longicornis, more data in our previous study. Among these ticks, 75% were adults, 20% were nymph and larva were 5% approximately, female were 60% and meal were 40%.

### Optimized Phi29 amplification efficacy of tick DNA

**Figure 2:**
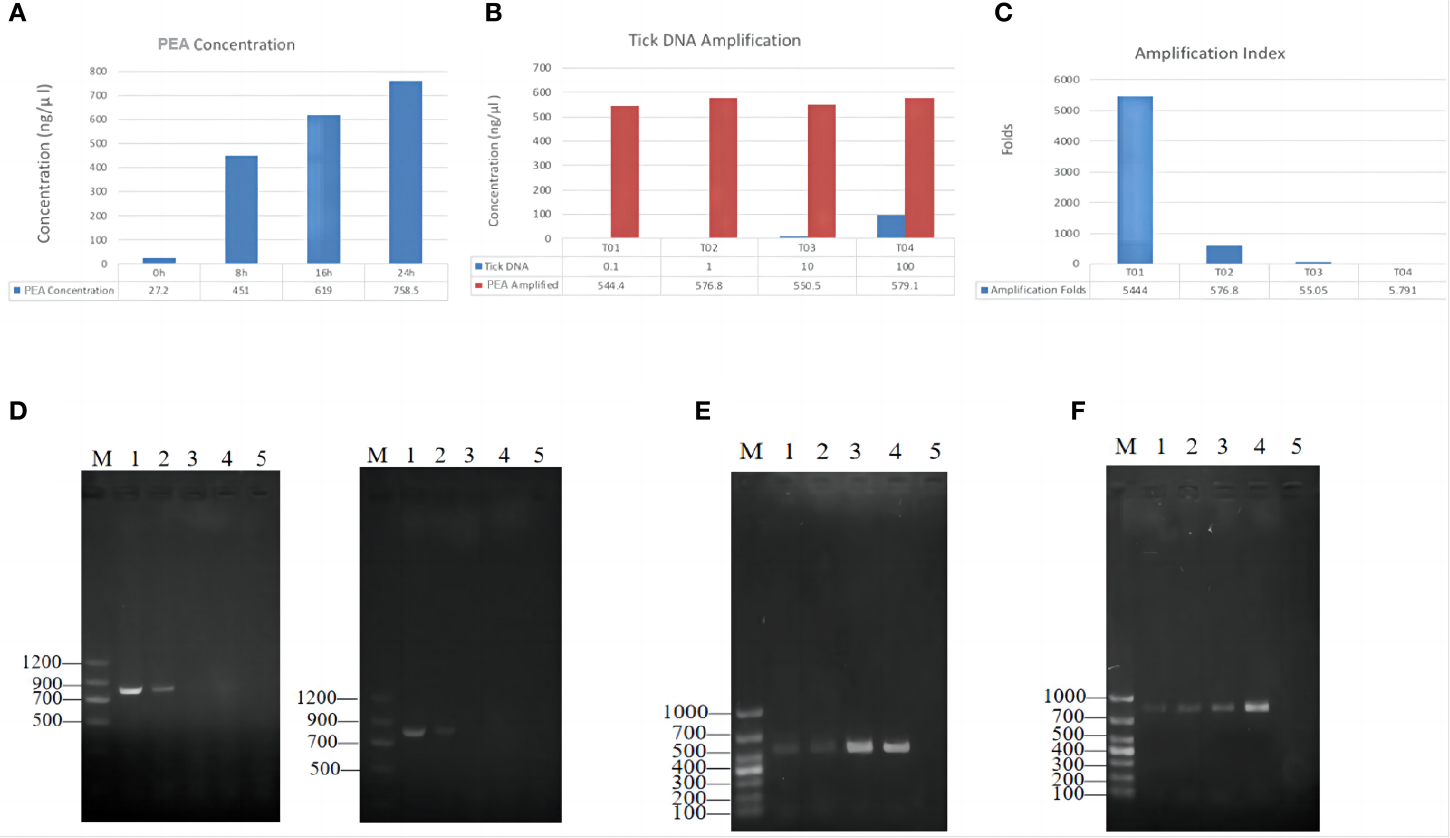
PEA amplification efficacy of single tick DNA. **(A)** Effects of incubation time on final PEA product concentration; Tick DNA was amplified for 8, 16 and 24 h, the product concentration was positively correlated with the incubation time. **(B)** Amplification index for different amount of starting DNA. **(C)** DNA increase folds after PEA. **(D)** Amplification of *ompB*, gene from original tick DNA with different template concentrations, original DNA (Left), PAE Amplified DNA (Right); M, marker, 1–4, represent diluted PEA product concentration of 100, 10, 1, and 0.1 ng/μl, 5, negative control. **(E)** Amplification of *ompA* gene from PEA products. M, marker, 1–2 represent original DNA, 3–4 represent PAE Amplified DNA, the concentration was 10, 100, 10, and 100 ng/μl, respectively, and 5 was negative control. **(F)** Amplification of *gltA* gene from PEA products. Gel lane were same as **(E)**.

Twenty ticks were used for testing the feasibility of the PEA assay for amplifying DNA. Using PCR with three pairs of primers, we detected *Rickettsia* in 12 out of 20 samples (Table 1). First, 10 ng/μl of the positive sample EDG241 was used for PEA. After amplification, the concentrations of the amplification products after incubation for 8, 16, and 24 h were 220, 680, and 700 ng/μl, respectively (Figure 2A). Thus, we selected the incubation time of 16 h for subsequent experiments. Second, original tick DNA (EDG241) was diluted to concentrations of 100, 10, 1, and 0.1 ng/μl before amplification by PEA, and the concentrations of the final products were 579.1, 550.5, 576.8 and 544.4 ng/μl, respectively. This indicates that the final concentrations did not differ significantly, even though the input DNA concentrations varied 1,000 times. The fold increases after amplification were calculated for these four starting concentrations by dividing the final concentration by the original concentration. The four tick DNA products were amplified 5,444, 576, 55, and 5.79-fold, respectively.

### Detection of *Rickettsia* DNA from the amplified products

We then tested whether PEA also improved pathogen DNA quantity with the same sample, EDG241. The original DNA and PEA-amplified DNA were diluted with ddH_2_O to 100, 10, and 1 ng/μl, and subsequently amplified with PCR using primers for the *gltA, ompA* and *ompB* gene. As shown in Figure 2, *Rickettsia* DNA could only be amplified from original tick DNA with concentrations of 100 and 10 ng/μl. In contrast, amplification from the PEA-amplified DNA was successful for samples with concentrations of 100 and 10, and 1 ng/μl. This result indicates that pathogen DNA as well as tick DNA were significantly amplified by PEA simultaneously. This high amplification efficacy of *Rickettsia* DNA indicates that total DNA insigle tick are amplified.

### Improved pathogen detection rate by PEA

**Figure 3:**
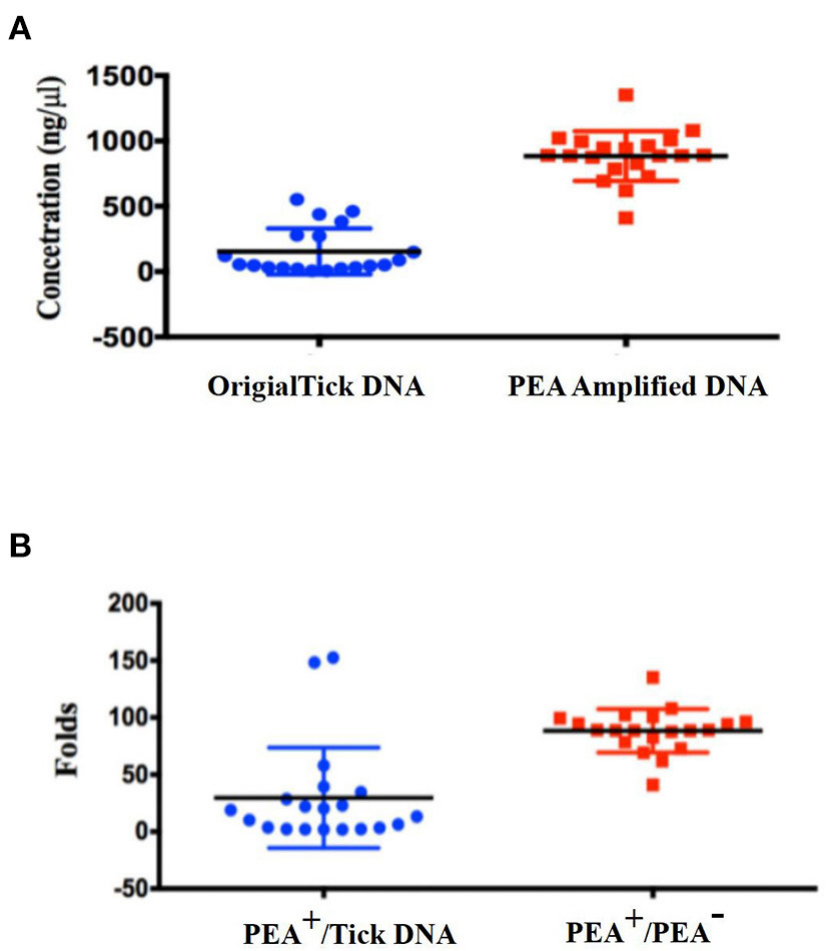
DNA concentrations and PEA amplification index for singular ticks. **(A)** Concentration distribution of original tick DNA and PEA amplified DNA; **(B)** PEA amplification index for single ticks.

After demonstrating that pathogen DNA was amplified unbiasedly, we tested whether the PEA could improve the positive rate of pathogen detection. Genomic DNA from 20 ticks selected according to the gender, host, and stage of the tick life cycle were extracted using a EasyPure Viral DNA/RNA Kit (TransGen Biotech, ER201-01, Beijing, China). The standard deviation of concentration of all 20 ticks was 176 ng/μl, but the DNA concentration varied significantly between the ticks, with final concentrations ranging from 6 to 552 ng/μl (Figure 3). We then subjected 10 ng of original tick DNA to PEA. The DNA was amplified to concentrations of > 700 ng/μl, with an average 200-fold increase. Amplified DNA was diluted and used for further amplification of the three genes of *Rickettsia*. As shown in Table 1, 12 of the 20 samples were positive for *Rickettsia* before amplification. After PEA amplification, 17 out of the 20 samples were positive. Our PEA assay could identify additional five samples, increasing the positivity rate by 25% (*P* = 0.02 <0.05), that probably because pathogen DNA after PEA was amplified, thus it was easier for detection.

### *Rickettsia* genotyping using PEA amplified DNA

**Figure 4:**
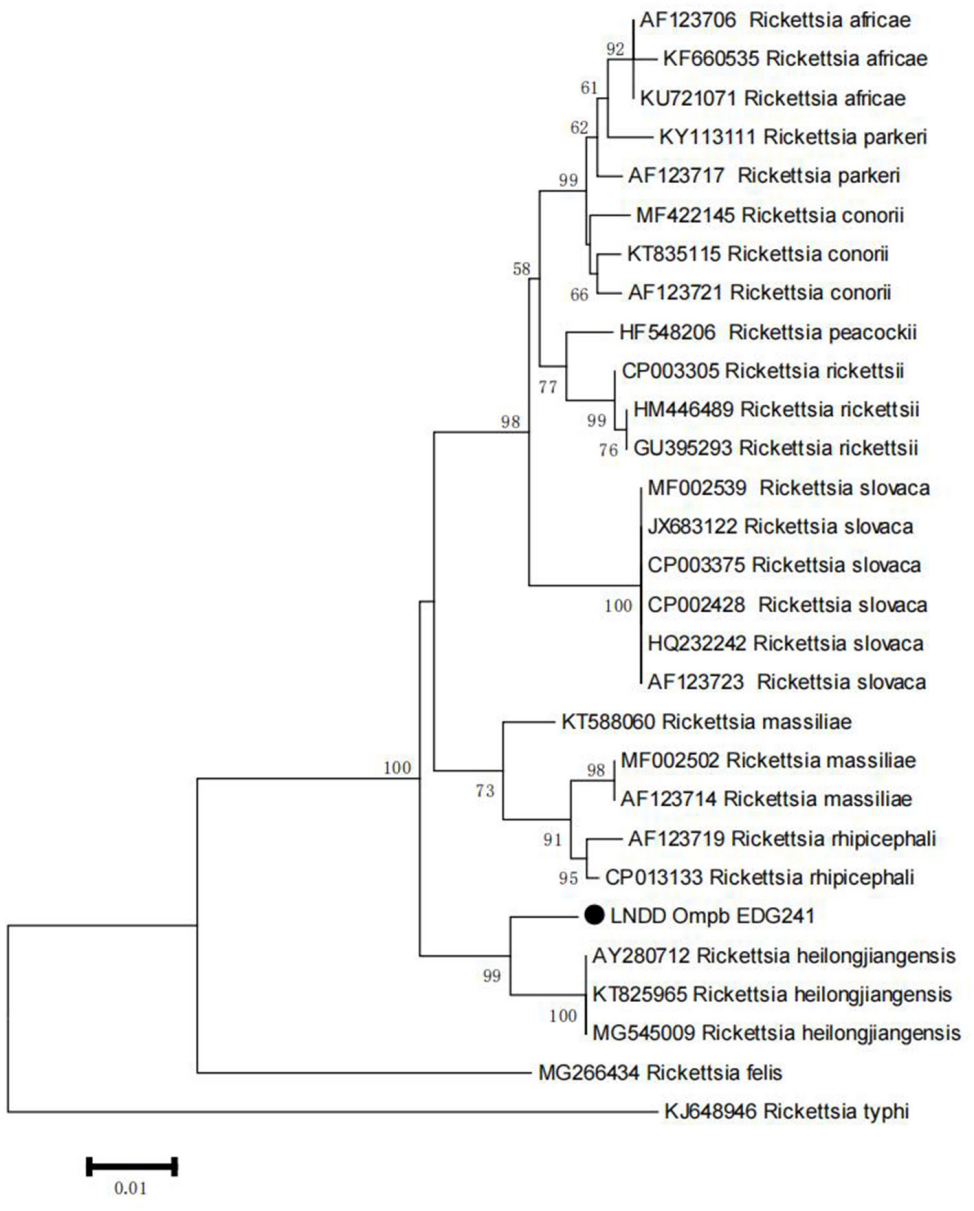
Phylogenetic tree of *Rickttsia* with *ompB* amplified from PEA products. The trees were constructed with sequence of ompB using maximum-likelihood (ML) algorithms implemented in the Molecular Evolutionary Genetics Analysis (MEGA) 7 software. Black dot represents sequence acquired from this study.

We sequenced and compared the PCR products that had been amplified either from PEA-amplified products or from original tick DNA. The sequences generated from PEA products were identical to those from original tick DNA. This is one of the most important requirements for genotyping, as any introduction of wrong bases will lead to incorrect genotyping. The high fidelity of PEA enables its use for pathogen genotyping. We selected *ompB* gene for the systematic analysis, the generated sequences of *ompB* were compared with those in the GenBank database, and a phylogenetic tree was constructed with NG modules implemented in MEGA software (Molecular Evolutionary Genetics Analysis, MEGA7, Auckland, New Zealand). We discussed the phylogenetic tree showed that the *Rickettsia* detected herein belongs to a novel genotype (Figure 4). According to the criteria for novel *Rickettsia* determination based on gene sequences, an isolate can be classified as a new *Rickettsia* genotype when amplifiable, ≥99.2% for *ompB*. Thus, we made a sequence alignment based on our sequenced genes *ompB*, between the *Rickettsia* detected in this study and R. heilongjiangensis. The results of the alignment with R. heilongjiangensis, the most homologous validated species, for which the percentages for the nucleotide identities were 98.63% for the *ompB* gene. Therefore, we temporarily determined that the detected *Rickettsiae* is a novel genotype as our previous study (17).

## Discussion

In this study, we developed and evaluated a new Phi29-based amplification assay, which we named PEA. Owing to the strong strand displacement activity of the Phi29 enzyme, the PEA is a powerful technique for amplification of trace amounts of DNA. The most important advantages of PEA are the production of long DNA products and the high efficiency and fidelity of the amplification. These advantages make PEA a very powerful tool, which will be useful for many applications.

PEA allows the amplification of DNA up to three orders of magnitude. In our present study, tick DNA was amplified up to 5,000 times. This yielded sufficient DNA for subsequent analysis. We also found that the final concentration of PEA products was correlated with the incubation time, but not with the original concentration of the template DNA; The starting DNA concentration ranged from 0.1 to 100 ng/μl, with a 1,000-fold difference; however, after 16 h of amplification, all final PEA products had concentrations around 550 ng/μl (Figure 2). Therefore, our PEA amplification technique provides a very efficient approach for generating template DNA for future use.

Notably, PEA is unbiased, a significant advantage over many other amplification technologies. As shown in this study, *Rickettsia* positive rate was improved by 30% post PEA. However, even with the high efficiency of PEA, the starting DNA concentration could not be reduced to < 1 ng/μl. Extremely low concentrations of starting template DNA (< 1 ng/μl) could lead to two critical problems: representativeness and amplification bias. We tested this by serial dilution of tick DNA. Although amplification products remained available, excessive dilution resulted in negative detection of *Rickettsia* DNA (data not shown). The reason for this might be that the low quantity of input DNA does not contain pathogen DNA, as it is present at lower concentration than tick DNA (20).

The feasibility of PEA for tick DNA amplification for pathogen detection and genotyping was then validated with ticks collected from Dandong, Liaoning province, China. As shown in Table 1, with PEA amplification, the detection rate of *Rickettsia* DNA in these samples was increased by 30%. This validated that PEA is very efficient not only for amplification of tick DNA but also for pathogen DNA. To further test the PEA efficacy, tick DNA was diluted, and *Rickettsia* DNA could be detected after PEA in samples with a starting concentration of as low as 1 ng/μl. However, without prior amplification, no *Rickettsia* DNA was detected (data not shown). This confirms that PEA amplifies both tick and pathogen DNA. Pathogen genotyping is significant for genetic analysis and for tracing the source of an outbreak. High amplification fidelity is key for correct genotyping (21). PEA results in DNA amplification up to three orders of magnitude, and any mutation occurring early during amplification could greatly change the sequence of the final product. We, therefore, compared the *gltA, ompA* and *ompB* sequences of original and PEA amplified products. No sequence differences were observed, indicating the high fidelity of PEA.

When developing the PEA assay for pathogen detection, we faced difficulty choosing an appropriate starting quantity of tick DNA. As the amount of pathogen DNA in tick DNA was unknown, it was difficult to define the optimal starting DNA^10^. Total DNA isolated from a single tick may vary greatly. As shown in Figure 3, the concentration of original tick DNA varied between 6 and 552 ng/μl, a 100-fold difference. In our study, the presence and amount of pathogen DNA in ticks also varies greatly. For the 20 ticks, 12 were positive by PCR before PEA amplification, and another six were positive when PEA was applied. We cannot know definitively that the other three samples were negative for *Rickettsia*. Alternative explanations are that the concentration of *Rickettsia* or *Rickettsia* DNA was too low to amplify detectable concentrations of *Rickettsia* DNA. We are currently trying to modify the PEA protocol to improve the concentration of pathogen DNA in the final PEA products.

## Conclusion

In the present study, we developed a PEA assay based on the unbiased strand displacement amplification activity of Phi29 DNA polymerase to amplify trace samples of DNA for ecological tick surveillance. With PEA, tick and tick-borne pathogen DNA could be unbiasedly amplified, resulting in sufficient sample DNA for subsequent detection and genotyping. This study demonstrates that PEA is a universal assay for the amplification of low amounts of DNA for genetic analysis.

## Data availability statement

The original contributions presented in the study are included in the article/supplementary material, further inquiries can be directed to the corresponding authors.

## Ethics statement

The animal study was reviewed and approved by Laboratory Animal Welfare Ethics Committee of Shenyang Agricultural University. Written informed consent was obtained from the owners for the participation of their animals in this study.

## Author contributions

ZC, HZ, and XH: conceived and designed the study. XZ, JC, PJ, HX, and QZ: performed the experiments and analyzed the data. XZ and ZC: draft and revised the manuscript. All authors contributed to the article and approved the submitted version.

## Funding

This work was supported by the State Key Program of National Natural Science of China (U1808202), China Postdoctoral Science Foundation (2021M692233), NSFC International (regional) Cooperation and Exchange Program (31961143024), the National Key Program for Infectious Disease of China (2018ZX10101002-002), and Key Program of Inner Mongolia (2019ZD006).

## Conflict of interest

The authors declare that the research was conducted in the absence of any commercial or financial relationships that could be construed as a potential conflict of interest.

## Publisher's note

All claims expressed in this article are solely those of the authors and do not necessarily represent those of their affiliated organizations, or those of the publisher, the editors and the reviewers. Any product that may be evaluated in this article, or claim that may be made by its manufacturer, is not guaranteed or endorsed by the publisher.
